# Identifying core global mental health professional competencies: A multi-sectoral perspective

**DOI:** 10.1017/gmh.2024.26

**Published:** 2024-02-22

**Authors:** Dimitar Karadzhov, Joanne Lee, George Hatton, Ross G. White, Laura Sharp, Abdul Jalloh, Julie Langan Martin

**Affiliations:** 1 School of Health & Wellbeing, College of Medical, Veterinary and Life Sciences, University of Glasgow, Glasgow, UK; 2 UNICEF, Eastern and Southern Africa Regional Office, Nairobi, Kenya; 3 School of Psychology, Queen’s University Belfast, Belfast, UK; 4 College of Medicine and Allied Health Sciences, University of Sierra Leone, Freetown, Sierra Leone

**Keywords:** Competence, Education, Training, Global mental health, Capacity

## Abstract

Concerned with sustainably alleviating mental distress and promoting the right to health worldwide, global mental health (GMH) is practised across various contexts spanning the humanitarian-development-peace nexus. The inherently intersectoral and multidisciplinary nature of GMH calls for competency frameworks and training programmes that embody diversity, decolonisation and multiprofessionalism. Existing competency frameworks have failed to capture the multi-sectoral, inter-professional nature of contemporary GMH practice. In response to these needs, a qualitative content analysis of relevant job advertisements was conducted to distil a comprehensive set of professional competencies in contemporary GMH practice. Approximately 200 distinct skills and competencies were extracted from 70 job advertisements and organised into four meta-dimensions: ‘*skills*’, ‘*sector*’, ‘*self*’ and ‘*subject*’. The first known systematic attempt at a multi-sectoral GMH competency framework, it offers a springboard for exploring vital yet overlooked professional competencies such as resilience, self-reflection, political skills and entrepreneurialism. On this basis, recommendations for building a competent, agile and effective GMH workforce with diversified and future-proof skillsets are proposed. The framework can also inform inter-professional training and curriculum design, and capacity-building initiatives aimed at early-career professional development, particularly in low- and middle-income settings.

## Impact statement

Professional competency frameworks play an important role in the education, training and continuous professional development of the diverse – multi-disciplinary and multi-professional – global mental health workforce. To reflect this diversity, a novel, multi-sectoral global mental health competency framework was developed from a job market analysis and a stakeholder consultation. This framework encompasses a range of job families such as advocacy, policy, service delivery, programme management, capacity development and research and teaching. Far from being a definitive list, the framework highlights the immense variety of interpersonal, technical, cognitive and knowledge-based competencies demanded from employers across sectors and roles. Alongside the well-recognised, core competencies such as collaboration, cultural sensitivity, integrity and intervention delivery, educators, trainers, managers and other leaders should develop trainees’ and professionals’ resilience and adaptability; creativity and curiosity; and entrepreneurial and reflective skills. The framework can foster inter-professional mobility and curriculum design, and inspire lifelong learning.

## Introduction

Global mental health (GMH) is a professionally diverse field of practice spanning multiple sectors and roles (Collins, 2020). It is practised across humanitarian, development and peace settings (World Health Organization, 2013, 2021). Because GMH is characterised by a diversity of disciplines, epistemologies, cultural perspectives and stakeholders, it defies easy categorisation (White et al., 2017; Rajabzadeh et al., 2021). It opposes an essentialist view of mental disorders and well-being, and instead centres on context, collaboration, empowerment, humility and power-shifting in its pursuit to sustainably alleviate mental distress and promote the right to health worldwide. This expansive and ambitious remit poses challenges to designing comprehensive training that caters to the wide range of GMH roles (Ng et al., 2016; Buzza et al., 2018; Acharya et al., 2024).

### The case for a multi-sectoral, multi-professional GMH competency framework

The intersectoral nature of GMH calls for competency frameworks and training programmes that embody diversity and multiprofessionalism (Di Ruggiero, 2022). Rather than narrowly viewed as a healthcare issue, GMH has also been construed as a human rights and a development issue (White et al., 2016). GMH is practised across contexts, with a significant presence within humanitarian and emergency settings. Roles often embrace the composite approach of *mental health and psychosocial support* (MHPSS), which is underpinned by inter- and multidisciplinary theoretical and conceptual frameworks and guidelines (Inter-Agency Standing Committee, 2007). This requires strong, diversified and transferable professional skills.

Accordingly, recommendations have been made to enhance GMH professional training via ‘[…] *inter-professional and trans-professional education that breaks down professional silos and enhances collaboration* […]’ (Fricchione et al., 2012, p. 53). Such training is instrumental to developing an agile, internationally and interprofessionally mobile, and collaborative workforce (Trowbridge et al., 2022). The ultimate goal, as Fricchione et al. (2012, p. 53) argue, should be to instil the skills, confidence and flexibility for professionals to access and scrutinise *‘global knowledge and experience*’ and apply them in solving ‘*local challenges*’.

Relatedly, Fernando (2017) highlights that *decolonising* the GMH curricula should be prioritised. Decolonisation refers to recognising and dismantling unjust and discriminatory power structures and practices maintained by dominant (e.g., Western) groups and cultures (Lewis et al., 2018). It seeks to legitimise and amplify the voices and experiences of historically oppressed groups. Decolonisation may be especially important for students from medical backgrounds, since it has been recognised that how mental health and mental ill health are taught within curricula has been heavily shaped by Eurocentric paradigms (Bracken et al., 2021). Cultural humility, structural competency and self-awareness are arguably vital in this process (Ng et al., 2016; Lewis et al., 2018; see Perkins et al., 2023, for a scoping review).

Several recent attempts have been made to systematise GMH competencies by either modifying related competency frameworks from adjacent disciplines (such as psychology and global health), or by creating bespoke frameworks or lists (e.g., Khoury, 2023). Notably, most of these have been focused on psychological service delivery (Institute of Medicine, 2013; International Association of Applied Psychology and International Union of Psychological Science, 2016; Buzza et al., 2018; World Health Organization and UNICEF, 2022), and (early-career) researchers (Thornicroft et al., 2012; Collins and Pringle, 2016; Ng et al., 2016; Merritt et al., 2019), and on specific interventions and settings such as humanitarian contexts (e.g., IFRC, 2016). To our knowledge, no unifying multi-sectoral GMH competency framework exists to date.

### Understanding employer needs

Concerns have been raised that competency lists developed by experts from curriculum reviews and other methods may not fully reflect contemporary professional practice (von Treuer and Reynolds, 2017). In contrast to traditional methodologies such as expert consultations and curricular reviews, job market analysis arguably offers a more objective, up-to-date and comprehensive overview of in-demand competencies (Brown et al., 2018; Keralis et al., 2018). It has been successfully used in fields such as global health to gauge employer expectations and boost students’ sector awareness (Brown et al., 2018; Keralis et al., 2018).

## Research context, aim and methods

The increased internationalisation of higher education, including the growing student diversity and mobility, and use of online distance learning, calls for effective, up-to-date and equitable approaches to supporting students’ professional development (Tran et al., 2023). In the context of a competitive global labour market, educators have a responsibility to maximise graduates’ employability, as well as help them acquire ‘[…] values, knowledge, attitudes and skills, dispositions and democratic principles […] to make a critically informed, responsible contribution to society’ (Robson and Wihlborg, 2019, p. 128). To help fulfil this responsibility, as part of a learning and teaching development project in a UK postgraduate taught GMH Master’s Degree programme, this study sought to identify a comprehensive set of in-demand GMH-related professional skills and competencies as indicated in relevant job descriptions and person specifications, across sectors and professional roles. A scoping job market analysis was carried out between September 2022 and January 2023.

Qualitative content analysis (QCA) was applied to extract competencies from the job advertisements. QCA is a systematic and transparent method for parsing textual data into distinct entities and generating concise informative summaries in the form of conceptual categories, systems or maps (Elo and Kyngäs, 2008, p. 108). We adopted an inclusive definition of competencies as encompassing ‘*an interplay of knowledge, capacities and skills, motives and affective dispositions*’ (Rieckmann, 2012, p. 129). Consistent with the World Health Organization (2017, p. ix), we recognise competencies are not fixed but ‘*dynamic and contextual*’.

### Data collection

We used purposive sampling techniques, which aim to maximise the sample’s richness, diversity and informativeness, while not claiming statistical generalisability (Suri, 2011). Job listings with rich descriptions of roles and candidate profiles were given preference, together with those reflecting common and feasible career paths for graduates (*intensity sampling*; Suri, 2011). Job listings for a wide range of GMH-related roles and sectors were selected (*maximum variation*; Suri, 2011). A pragmatic mix of generic (e.g., www.linkedin.com; https://uk.indeed.com/), sector-specific (e.g., https://unjobs.org; https://www.charityjob.co.uk; (https://www.jobs.nhs.uk/); https://www.jobs.ac.uk; https://cbm-global.org/) and country- and region-specific (e.g., https://ngojobsinafrica.com; https://www.ghanacurrentjobs.com) job sites was searched. To maximise retrieval, broad subject-, sector- and post-related search terms were used (e.g., ‘GMH’, ‘psychology’, ‘clinical’, ‘policy’, ‘MHPSS’, ‘counsellor’, ‘capacity’ and ‘research’). Initially, 250 advertisements were screened for relevance; from these, 50 were selected using the criteria above and sorted into job families. Another 120 advertisements were then screened in search of advertisements from underrepresented job families. This resulted in another 20 advertisements being added to the final sample.

At 70 advertisements, saturation was reached – analysing additional advertisements did not yield substantially new competencies (Morse, 2015).

To track and ensure a multi-sectoral scope, the researchers categorised the 70 advertisements into several job families: advocacy (7; 10%); capacity development (8; 11%); policy (7; 10%); programme implementation, management and evaluation (12; 17%); research (15; 21%); clinical, psychological and psychosocial service delivery (17; 24%) and teaching (4; 6%). The jobs were based in the United Kingdom (34); the African continent (17, including Ethiopia, Nigeria, Tanzania, Zanzibar, South Sudan, Senegal, Kenya, Rwanda, Uganda, Kuwait, Ghana and Mali); multiple countries (4); other European countries (3); the Americas (3); New Zealand (2) and remote or other (7). A wide range of employers and sectors were represented, including humanitarian aid organisations, charities and other non-profit organisations, government agencies, higher education institutions, international NGOs and the private sector. The sample featured a mix of positions suitable for graduates and early-career professionals, mid-career professionals and highly specialised and/or senior roles. The aim was to include both foundational, entry-level competencies and more advanced and aspirational ones.

### Data analysis

The advertisements were exported into the qualitative data analysis software programme, NVivo 12 (https://support.qsrinternational.com/s/), where the person specifications, main responsibilities, required qualifications and employer information were manually analysed using QCA by the first and third authors. The purposive sampling and QCA aimed to map the *range* of relevant competencies; therefore, frequencies were not calculated. First, the advertisements were read and re-read line-by-line, following which codes corresponding to individual competencies were ascribed to short phrases or sentences (Elo and Kyngäs, 2008). Then, the long list of initial codes was re-examined, and codes were grouped into sub-categories based on similarities. The sub-categories were then clustered into a smaller number of higher-level, more abstract meaning units called categories (see Table 1, for an example of the coding process). The third author conducted the initial coding of all advertisements, after which the first author reviewed this initial analysis for logical consistency and carried out further analysis, as required. The two authors met frequently to discuss coding decisions and resolve any discrepancies. The third author is a health researcher and recent MSc GMH graduate, who had received training in qualitative data analysis. The first author is a researcher with expertise in public health, GMH and qualitative methodologies.

**Table 1.** Example of the qualitative content analysis coding process

Ad excerpt	Code	Sub-category	Category
‘*Flexibility to respond to changing and challenging circumstances*’	Flexibility and adaptability	Personal resources	Self
‘*Emotionally resilient*’	Resilience and grit		
‘*Can remain calm under pressure*’	Composure		
‘*Positive and flexible attitude to work*’	Positivity		

The final stage of the QCA entailed the creation of a model or a conceptual map (Elo and Kyngäs, 2008; Elo et al., 2014) – an accessible four-dimensional competency framework. To increase trustworthiness, the first iteration of the framework was sent to 17 potential users of the framework, including seven experts from Ukraine, Sierra Leone, Uganda, Nigeria, Egypt and the United Kingdom, and 10 recent MSc GMH graduates. They were asked to advise whether the framework and language used were clear, understandable and usable (Elo et al., 2014). Their feedback helped identify and remove jargon and ambiguity, enhancing its international applicability.

## Results

**Figure 1.**
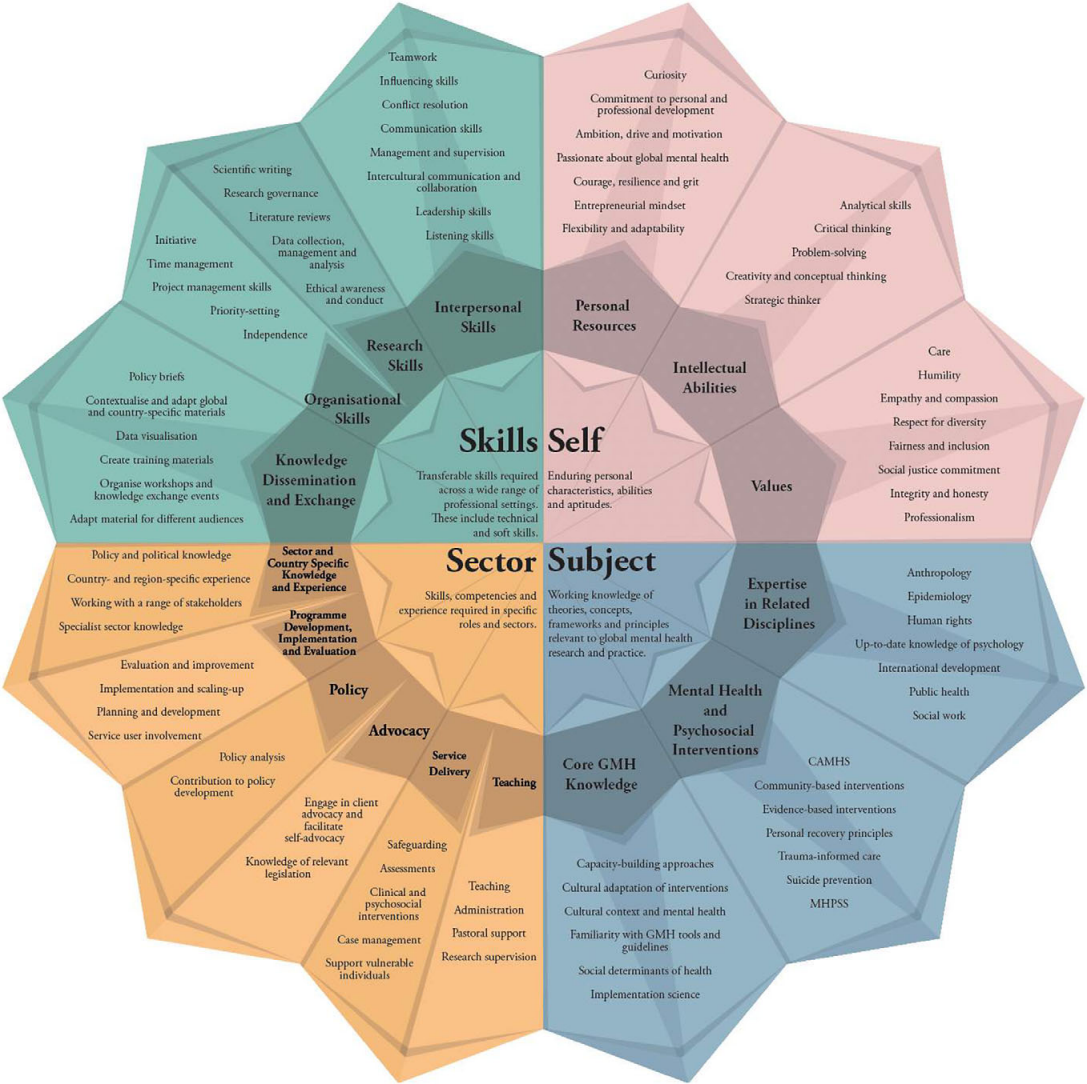
The ‘4S’ Multi-Sectoral GMH Competency Framework. *Note:* The list of competencies is not exhaustive. CAMHS - child and adolescent mental health services; GMH - global mental health; MHPSS - mental health and psychosocial support.

Approximately 200 distinct skills and competencies were derived from the 70 job advertisements (see Supplementary Material for the full list). Four meta-level categories were found to reasonably accommodate the QCA codes and sub-categories (Figure 1):

- *‘Self’* – enduring personal characteristics, abilities and aptitudes;
- *‘Skills’* – transferable skills required across a wide range of professional settings, including technical and interpersonal skills;
- *‘Sector’* – skills, competencies and experience required in specific roles and sectors;
- *‘Subject’* – working knowledge of theories, concepts, frameworks and principles relevant to GMH research and practice.

### Self: Intellectual abilities, personal resources and values

This category encompasses three sub-categories: intellectual abilities, personal resources and values. *Intellectual abilities* are highly prevalent within job descriptions, and refer to cognitive abilities pertaining to analysing, synthesising and interpreting information and generating ideas and solutions. In addition to the quintessential skills of analytical and critical thinking, and problem-solving, employers placed a strong emphasis on creativity, idea generation and innovation. These were especially common in capacity development, service delivery and research roles. These attributes were often described as being open to change and ‘thriving’ in solving problems:

Should thrive in solving problems and producing pragmatic solutions (Regional Scaling Coordinator, War Child Holland).

Research never goes exactly according to plan, and the Research Officer must approach the problems that will inevitably arise with patience and creativity […] Creativity and an ability to think outside the box to conceptualize projects and implementation strategies (Research Officer – South Sudan, Forcier Consulting).

Suggests creative improvements and better ways of working (Research Coordinator, The MHPSS Collaborative).

Creative and proactive problem solver (Social Worker – Ghana, International Justice Mission).

The second sub-category, *personal resources*, is a highly heterogeneous cluster of traits and competencies that promote coping, resilience and thriving in the workplace (Kasler et al., 2017; See Figure 1). The most frequent ones across sectors and roles were: (a) flexibility, adaptability and openness to change; (b) resilience and grit and (c) ambition, drive and determination. Specifically, there was a strong focus on working effectively amidst changing, challenging and unclear circumstances. Notably, these were not reserved for humanitarian emergency jobs but were also required in service delivery roles in peace settings:

Strong self-starter, able to take initiative and adapt to changing circumstances and priorities (Psychological Counsellor – Ethiopia, Save the Children).

Shifts tasks, roles and priorities to perform effectively under changing or unclear conditions (Research Coordinator, The MHPSS Collaborative).

Managing self: Displays grit, courage, resilience […] (Senior Policy Advisor, Mental Health and Wellbeing Commission).

Less commonly mentioned but noteworthy were self-discipline, curiosity, accountability, composure, courage, entrepreneurialism, among others – for example: ‘*entrepreneurial mindset*’ (Healthcare Partnerships & Service Integration – Kenya, Zipline); ‘*start-up mentality*’ (Lead Clinical Psychologist – Nigeria, Reliance Health); ‘*stand up and challenge decisions*’ (Advocacy Worker, Think Care Careers); ‘*we are courageous and speak up about what is important to people*’ (Senior Policy Advisor – New Zealand, Te Hiringa Mahara).

Finally, *values* are a sub-category that is distinct from intellectual abilities or traits and attributes promoting coping amidst change and adversity. This sub-category was derived from coding both the personal specifications and the employer information sections, and was prevalent across job families, including service delivery, advocacy and policy. A wide range of values were expected – including respect for diversity; integrity and professionalism; social justice commitment and fairness and inclusion:

Knowledge and commitment to anti-racist and inclusive practices. […] Demonstrable commitment to upholding the rights of people who face disadvantage or discrimination (Independent Advocate, Gaddum Advocacy).

Act in a way that acknowledges and recognizes people’s expressed beliefs, preferences and choices (Assistant Psychologist, NHS Wales).

### Skills: Interpersonal skills, research skills and organisational skills

This category accommodates the vast range of transferable skills – including *interpersonal skills, organisational skills* and *research skills* – that were the backbone of virtually all advertisements (see Figure 1). In addition to quintessential employability skills such as teamwork, time management, initiative and leadership, the analysis highlighted several more advanced and GMH-specific competencies, particularly *intercultural communication and collaboration*; *influencing skills*; *negotiation skills* and *networking* and *relationship-building* within and across sectors:

Excellent relationship building and influencing skills (Senior Policy Officer – London, NHS).

The role involves lobbying and developing understanding of the interventions and tools, organize and participate in adaption and contextualizing workshops and conducting online or face-to-face training with stakeholders and partners based in different countries across the region (Regional Scaling Coordinator, War Child Holland).

Work closely with the Psychological Intervention Specialist to conduct consultations with multi-sector stakeholders, community-based organizations, international NGOs (Psychological Intervention Researcher, UNICEF).

Respectfully engage with partners and fellow staff members from different cultural backgrounds (Research Officer – South Sudan, Forcier).

Next, *research skills* represent a highly varied cluster comprising technical (e.g., data collection and analysis, information literacy); scientific communication (e.g., report writing, grant proposals and translating research into practical recommendations) and interpersonal (e.g., ethical awareness, political sensitivity) skills. Importantly, research skills were found across job families, including service delivery, policy, capacity development and intervention evaluation.

Furthermore, several of the advertisements expected candidates to engage in various knowledge dissemination and exchange activities such as policy briefs, stakeholder dialogues, training materials and locally adapted guidelines:

Preparation of accessible high quality reports on complex issues – for example policy positions, national consultations and member briefings (Senior Policy Officer – London, NHS).

Assist in the creation of training material and reports (Intern – Peru, Innovations for Poverty Action).

Adapt existing training packages to the current context and different needs (MHPSS Technical Advisor, Red Cross).

### Sector: Sector- and country-specific competencies and experience

The *sector* category houses competencies that are highly specific to the included job families – for instance, carrying out psychological assessments, cross-sectoral liaison, client advocacy, capacity and needs assessment, programme scale-up and policy analysis and development (see Figure 1):

Outstanding capacity to understand the country context, portfolio, and overall programmatic needs (Mental Health Technical Advisor, International Rescue Committee).

To work alongside and ensure active service user participation in all aspects of work, including design, implementation and monitoring of activities (Mental Health Worker (Dual Diagnosis), Richmond Borough Mind).

*Programme development, implementation and evaluation skills*, in particular, encompass collecting data on programme outcomes, contextualising and adapting materials, supporting the scaling-up of evidence-based interventions, training stakeholders, involving service-users and others (see Supplementary Material).

Commonly required across sectors and roles was the ability to *work with a wide range of stakeholders*, including governmental and non-governmental agencies, professional groups, service-users, communities and donors. Examples of specific professional activities and skills were stakeholder consultation, coordination, motivation and persuasion, in addition to partnership-building. Those often entailed demonstrable knowledge and understanding of key stakeholders at global and regional levels.

Relatedly, knowledge of relevant systems, legislation and policies was also commonly expected, including global frameworks and in-country policies:

Knowledge of international policies, laws and mandates as pertaining to pesticides and suicides (WHO Consultant).

Finally, several jobs, mostly in capacity development, required experience of working in a low- or middle-income country (LMIC), in war-affected regions, or in a country different from one’s own.

### Subject: Core GMH knowledge and knowledge of related disciplines

Finally, this category represents the knowledge-based competencies that were either essential or desirable candidate characteristics (see Figure 1). The analysis distinguishes between *core GMH knowledge* (e.g., knowledge of scaling-up approaches and of cultural adaptation of interventions) and broader knowledge of *mental health and psychosocial interventions and theories.*

Partly due to the inclusive, multi-sectoral scope of the framework, employers commonly expected up-to-date knowledge of, or expertise in, related disciplines such as human rights, international development, social work, public health, anthropology and epidemiology.

## Discussion

This study aimed to scope the international GMH job market and extract and synthesise in-demand competencies from relevant job advertisements in order to develop a novel, multi-sectoral GMH competency framework. The framework showcases the diversity of sectors, disciplines and transferable skills relevant to contemporary GMH practice, and highlights areas for workforce development (Fricchione et al., 2012; Ng et al., 2016).

### Research skills

A noteworthy finding of the job market analysis was the presence of research skills in non-research positions such as policy, programme management and psychological service delivery. Research skills training is essential to building capacity for GMH, particularly in LMICs (Wainberg et al., 2017; Okewole et al., 2020). Adoption of research competencies has been shown to boost personal and professional growth (Okewole et al., 2020), as well as increasing capacity for implementing evidence-based care (Thornicroft et al., 2012; Wainberg et al., 2017; Merritt et al., 2019). As Abu-Zaid (2014) shows, few medical students show interest in undergraduate research engagement as well as research-based careers mainly due to factors such as insufficient exposure to scientific research early in education, unwillingness to prolong medical training, personal preference and failure to understand the importance of having research skills in general practice. Thus, building a positive attitude towards research remains a priority (Merritt et al., 2019). Merritt et al. (2019) highlight the value of improving research skills in those working in the GMH field in LMICs countries to help rebalance historical underinvestment. The current job market analysis also underscores the need to scale up access to professional development programmes for GMH professionals focusing on knowledge-to-practice translation (Wainberg et al., 2017).

### Communication and political skills

While an array of communication skills have invariably featured in existing competency frameworks (Buzza et al., 2018; Merritt et al., 2019), the current job market analysis shines a light on a broader spectrum of communication and political skills, including some less commonly emphasised skills such as conflict resolution and influencing skills, together with policy and political knowledge and sensitivity. Multi-stakeholder collaboration, negotiation and consensus-building are indispensable in many GMH-related roles, particularly in the policy, advocacy, capacity-building and healthcare management sectors (Ng et al., 2016; Iemmi, 2022). Negotiation, specifically, has been highlighted as an emotionally demanding skill; hence, its close links to leadership, emotional intelligence and resilience (Higazee and Gab Allah, 2022). This signals the need to embed such training in GMH programmes – for instance, in the form of simulation and role-play activities (Higazee and Gab Allah, 2022).

### Emphasis on higher-order competencies

A distinctive finding of the current analysis – over and above existing frameworks – is the strong emphasis on dispositional and motivational characteristics and complex intellectual skills such as creativity, strategic thinking, perseverance and curiosity. This tendency was also observed in Keralis et al.’s (2018) market analysis of global health jobs. As Jardim (2021, p. 1) argues, ‘[c]*ognitive and technical skills are not sufficient to face the professional challenges of the current digital and global world* […]’. Some of these competencies (e.g., resilience, entrepreneurial mindset, courage, curiosity, perseverance, passion and adaptability) may be difficult to measure or evaluate, and have therefore remained under-investigated in the professional skills development literature (Jogerst et al., 2015; von Treuer and Reynolds, 2017). We nevertheless propose they remain important, and often integral, to effective professional activity in the GMH field.

### Resilience and adaptability

The inclusion of resilience and adaptability in the current framework is not surprising given the challenging, resource-constraint contexts of much of GMH practice, together with the often-stigmatised, underfunded and neglected issues professionals in this field seek to redress. GMH professionals operate in ideologically and professionally contested and politically and economically unstable settings, and are therefore required to be adaptable, resilient and persevering; they are required to be pathfinders (White et al., 2017; Dean et al., 2020). Most recently, the COVID-19 pandemic has demonstrated, and reinvigorated interest in, the importance of fostering resilience in healthcare and aid workers (Dean et al., 2020; Young et al., 2022). The capacities for resilience and adaptability have been identified as core to sustainable development (Rieckmann, 2012; Brundiers et al., 2021). There is a strong case, therefore, that such practices should be embedded in GMH curricula.

As Matheson et al. (2016) note, it would be fruitful to explore the practices and characteristics of resilient professionals, and assess the extent to which those could be trained and taught. Among the resilience-promoting workplace interventions and behaviours suggested by Matheson et al.’s (2016) participants are exposure to challenging situations, peer learning and mindfulness. All of these could be adapted to the classroom. Learners and trainees should also be provided with opportunities for experiential learning through internships, mentorship, site visits and engagement with communities of practice.

Importantly, traits and skills such as resilience and adaptability can be viewed as characteristics of organisational culture and structures and team dynamics, in the context of broader external forces, not merely as individual attributes (Dean et al., 2020; Masten and Motti-Stefanidi, 2020). This is echoed by Eichbaum’s (2015) concern that an individualistic view of global health competencies promotes an individualistic approach to learning and assessment, which may be at odds with the interactional, dynamic way such competencies are acquired and distributed within low-resource settings. We therefore encourage readers to critically examine the applicability of the Multi-Sectoral GMH Competency Framework at the organisational, sector, team and community levels.

### Entrepreneurialism and GMH

Entrepreneurial skills and aptitudes (such as a start-up mentality, strategic thinking, perseverance, persuasion, optimism, generativity, drive and flexibility) also emerged as important. However, these have remained overlooked in existing competency frameworks. Acute global crises such as COVID-19, together with rapid technological advancements such as the rise of digital healthcare and artificial intelligence, have provided fertile ground for *global entrepreneurship*, including in LMICs (Mishra and Pandey, 2023). We hereby argue that an entrepreneurial lens offers an opportunity to reinvigorate GMH training by increasing the focus on developing trainees’ creative, enterprising and leadership capabilities (Kidd et al., 2015; Tang et al., 2018). We urge educators, trainers, managers and other leaders to explore ways to cultivate entrepreneurial skills in the GMH workforce – for example, by leveraging international partnerships, internships, mentorship and alumni engagement (Colombelli et al., 2022).

### Suggested applications of the framework

By design, the framework incorporates a considerable number of competencies across sectors, job titles and career stages. Consequently, students, trainees and professionals are *not* expected to be proficient at all of them. Rather, the framework can be used to identify training needs, articulate already-acquired skills, aid career planning and mobility and promote self-awareness and reflection, as well as lifelong learning (Jogerst et al., 2015; Okewole et al., 2020). To both prospective students and employers, it can demonstrate the breadth and transferability of skills acquired in GMH programmes and training (Acharya et al., 2024). Relatedly, it can be used in programme advertisement and candidate selection so that candidates are aware of the scope and remit of job opportunities post-qualification. The framework can also aid educators in designing programmes aligned with the job market, including collaborative, interprofessional training programmes (Rowthorn and Olsen, 2014; Okewole et al., 2020; Acharya et al., 2024).

A key challenge for educators and trainers is how to foster and assess these competencies (Schleiff et al., 2020). As Vikram Patel, Chair of the Department of Global Health and Social Medicine at Harvard Medical School, aptly notes, adopting a competency-based approach to education and training goes hand in hand with introducing novel forms of assessment and teaching that resemble the specifics and challenges of doing GMH in the field (Institute of Medicine, 2013). Many of the competencies identified in the framework can directly inform creative authentic assessments and training opportunities, and equip trainees to undertake the decolonisation process. Techniques such as scenario-based learning, peer feedback and situational judgement tests have shown promise in this area (Wroe et al., 2017; Aylott et al., 2023). Many of the identified attitudinal and technical competencies are likely best developed in the field (Eichbaum, 2017). To allow for students’ and trainees’ exposure to authentic professional and cultural settings, multi-country partnerships, particularly between high-income and LMICs, should be established (Marienfeld et al., 2024). Assessment – particularly of the more situational and relational competencies such as intercultural communication, humility, influencing skills, conflict resolution and social justice awareness – should include more naturalistic, contextual and reflexive approaches such as role plays, observation and reflective field reports, and ideally involve co-assessors from diverse socio-cultural backgrounds (Eichbaum, 2015, 2017).

Above all, competency frameworks developed in high-income, Western settings by mostly Western researchers such as the current framework should be adapted to local settings critically, ethically and equitably (Schleiff et al., 2021). The ‘blind’ imposition of Western values and concepts without prioritising context-specific training needs, learning preferences, cultural norms and worldviews exemplifies colonial practice and amounts to social injustice (Pritchard et al., 2023). Individuals working in the field of GMH have a responsibility to be familiar with and continuously challenge the normalisation of embedded colonial assumptions and processes. Understanding how these histories shape current practice provides opportunities for practitioners to apply their own values and knowledge to help redress power imbalances and progress the decolonisation process across practice (Pritchard et al., 2023; Sridhar et al., 2023). The inclusion of values (including respect for diversity, social justice commitment and fairness) as a competency meta-dimension underscores their centrality to effective, equitable and sustainable GMH practice (Kohrt et al., 2016). Guided (self-)reflection, exposure to different cultural perspectives and experiences and knowledge of how power, inequality and oppression have operated historically to marginalise communities in LMICs remain vital (Sridhar et al., 2023). Self-awareness, humility, resilience, curiosity and courage – all distilled in the current framework – are fundamental personal attributes in undertaking this work (Ventres, 2019).

### Limitations and future directions

The framework discussed in this article is limited in several ways. First, it is only one of several possible conceptual representations of the competencies extracted from the advertisements. The boundaries between the dimensions and sub-dimensions are only tentative, however, and do not determine the order or priority in which these competencies should be acquired. Second, the sample is relatively small, and many regions and job roles have remained underrepresented; in particular, advertisements that do not use traditional mental health job titles but are within the GMH scope, for example, child protection and occupational therapy roles. Third, the framework was initially designed to boost the employability of English-speaking GMH graduates in the UK, and due to its reliance on job advertisements as the data sources, one could argue that it represents an *employability framework* more so than a competency framework. The adopted definition of *competencies* was purposefully broad, and we acknowledge alternative definitions exist (von Treuer and Reynolds, 2017). Finally, and fundamentally, although effort was made to include advertisements from different countries, the framework largely reflects a Eurocentric perspective. Global North jobs and employers, who may unintentionally prioritise Western values and epistemologies, were oversampled – creating bias in the current study.

Priority areas for follow-up research include validating the framework across LMICs and diverse communities of practice (e.g., see Zwanikken et al., 2014); mapping competency gaps among trainees and professionals in low-resource settings, and redressing structural barriers to the acquisition of required competencies among these groups (Hansoti et al., 2021; Schleiff et al., 2021). Further work is also warranted into translating the framework into training guides that can be adapted for different sectors, professional groups and cultural settings, as well as into feasible curriculum learning objectives that can be adequately assessed using creative and contextualised assessment techniques (Schleiff et al., 2020; Brundiers et al., 2021). Notwithstanding the aforementioned limitations, the current study has strong potential for advancing and democratising professional development in the field.

## Supporting information

Karadzhov et al. supplementary material Karadzhov et al. supplementary material

## Data Availability

The data that support the findings of this study (i.e., copies from the job advertisements that informed the analysis) are available from the corresponding author, D.K., upon request.

## Supplementary material

The supplementary material for this article can be found at https://doi.org/10.1017/gmh.2024.26.

## References

[r1] Abu-Zaid A (2014) Research skills: The neglected competency in tomorrow’s 21st-century doctors. Perspectives on Medical Education 3(1), 63–65. 10.1007/s40037-013-0087-7. 24092545 PMC3889998

[r2] Acharya B, Buzza C, Guo J, Basnet M, Hung E and Van Dyke C (2024) Developing a global mental health training curriculum. In Acharya B and Becker AE (eds.), Global Mental Health Training and Practice. New York: Routledge, pp. 81–95.

[r3] Aylott LM, Finn GM and Tiffin PA (2023) Assessing professionalism in mental health clinicians: Development and validation of a situational judgement test. BJPsych Open 9(6), e213. 10.1192/bjo.2023.582. 37955048 PMC10753968

[r4] Bracken P, Fernando S, Alsaraf S, Creed M, Double D, Gilberthorpe T and Timimi S (2021) Decolonising the medical curriculum: Psychiatry faces particular challenges. Anthropology & Medicine 28(4), 420–428. 10.1080/13648470.2021.1949892. 34282672

[r5] Brown HA, Mulherin P, Ferrara WC, Humphrey ME, Vera A and Hall JW (2018) Using future employers’ expectations to inform global health fellowship curricula. Journal of Graduate Medical Education 10(5), 517–521. 10.4300/JGME-D-18-00348.1. 30386476 PMC6194889

[r6] Brundiers K, Barth M, Cebrián G, Cohen M, Diaz L, Doucette-Remington S, Dripps W, Habron G, Harré N, Jarchow M, Losch K, Michel J, Mochizuki Y, Rieckmann M, Parnell R, Walker P and Zint M (2021) Key competencies in sustainability in higher education—Toward an agreed-upon reference framework. Sustainability Science 16, 13–29. 10.1007/s11625-020-00838-2.

[r7] Buzza C, Fiskin A, Campbell J, Guo J, Izenberg J, Kamholz B, Hung E and Acharya B (2018) Competencies for global mental health: Developing training objectives for a post-graduate fellowship for psychiatrists. Annals of Global Health 84(4), 717–726. 10.29024/aogh.2382. 30779522 PMC6748267

[r8] Collins PY (2020) What is global mental health? World Psychiatry 19(3), 265. 10.1002/wps.20728. 32931115 PMC7491634

[r9] Collins PY and Pringle BA (2016) Building a global mental health research workforce: Perspectives from the National Institute of Mental Health. Academic Psychiatry 40, 723–726. 10.1007/s40596-015-0453-3. 26586615 PMC4873457

[r10] Colombelli A, Loccisano S, Panelli A, Pennisi OAM and Serraino F (2022) Entrepreneurship education: The effects of challenge-based learning on the entrepreneurial mindset of university students. Administrative Sciences 12(1), 10. 10.3390/admsci12010010.

[r11] Dean L, Cooper J, Wurie H, Kollie K, Raven J, Tolhurst R, MacGregor H, Hawkins K, Theobald S and Mansaray B (2020) Psychological resilience, fragility and the health workforce: Lessons on pandemic preparedness from Liberia and Sierra Leone. BMJ Global Health 5(9), e002873. 10.1136/bmjgh-2020-002873. PMC7523196 32988928

[r12] Di Ruggiero E (2022) Addressing mental health through intersectoral action in the context of COVID-19 and the 2030 agenda for sustainable development. Global Health Promotion 29(3), 3–4. 10.1177/17579759221122710. 36281928

[r13] Eichbaum Q (2015) The problem with competencies in global health education. Academic Medicine 90(4), 414–417. 10.1097/ACM.0000000000000665. 25692558

[r14] Eichbaum Q (2017) Acquired and participatory competencies in health professions education: Definition and assessment in global health. Academic Medicine 92(4), 468–474. 10.1097/ACM.0000000000001382. 27603041

[r15] Elo S, Kääriäinen M, Kanste O, Pölkki T, Utriainen K and Kyngäs H (2014) Qualitative content analysis: A focus on trustworthiness. SAGE Open 4(1), 1–10. 10.1177/2158244014522633.

[r16] Elo S and Kyngäs H (2008) The qualitative content analysis process. Journal of Advanced Nursing 62(1), 107–115. 10.1111/j.1365-2648.2007.04569.x. 18352969

[r17] Fernando S (2017) Institutional Racism in Psychiatry and Clinical Psychology. London: Palgrave Macmillan.

[r18] Fricchione GL, Borba CP, Alem A, Shibr T, Carney JR and Henderson DC (2012) Capacity building in global mental health: Professional training. Harvard Review of Psychiatry 20(1), 47–57. 10.3109/10673229.2012.655211. 22335182 PMC3335114

[r20] Hansoti B, Hahn E, Dolive C, Akridge A, Atwell M, Mishra A and Schleiff M (2021) Training global health leaders: A critical review of competency gaps. Annals of Global Health 87(1), 65. 10.5334/aogh.3260. 34307068 PMC8284503

[r21] Higazee MZA and Gab Allah AR (2022) The relationship between the political skills and negotiation behaviors of front‐line nursing managers. Nursing Forum 57(6), 1240–1248. 10.1111/nuf.12772. 35781281

[r22] Iemmi V (2022) Establishing political priority for global mental health: A qualitative policy analysis. Health Policy and Planning 37(8), 1012–1024. 10.1093/heapol/czac046. 35763373 PMC9384251

[r23] IFRC (2016) Competency framework: Psychosocial support delegates in emergencies. Available at https://pscentre.org/wp-content/uploads/2018/10/Final-versionCompetency-Framework-September-16.pdf (accessed 31 August 2023).

[r24] Institute of Medicine (2013) Strengthening Human Resources through Development of Candidate Core Competencies for Mental, Neurological, and Substance Use Disorders in Sub-Saharan Africa: Workshop Summary. Washington, DC: The National Academies Press. 10.17226/18348. 24199264

[r68] Inter-Agency Standing Committee (2007) IASC guidelines on mental health and psychosocial support in emergency settings. Available at https://interagencystandingcommittee.org/iasc-task-force-mental-health-and-psychosocial-support-emergency-settings/iasc-guidelines-mental-health-and-psychosocial-support-emergency-settings-2007 10.1080/09540261.2022.2147420 36502397

[r25] International Association of Applied Psychology & International Union of Psychological Science (2016) International declaration of core competencies in professional psychology. Available at https://www.iupsys.net/wp-content/uploads/2021/09/the-international-declaration-on-core-competences-in-professional-psychology-1.pdf (accessed 31 August 2023).

[r26] Jardim J (2021) Entrepreneurial skills to be successful in the global and digital world: Proposal for a frame of reference for entrepreneurial education. Education Sciences 11(7), 356. 10.3390/educsci11070356.

[r27] Jogerst K, Callender B, Adams V, Evert J, Fields E, Hall T, Olsen J, Rowthorn V, Rudy S, Shen J, Simon L, Torres H, Velji A and Wilson LL (2015) Identifying interprofessional global health competencies for 21st-century health professionals. Annals of Global Health 81(2), 239–247. 10.1016/j.aogh.2015.03.006. 26088089

[r28] Kasler J, Zysberg L and Harel N (2017) Hopes for the future: Demographic and personal resources associated with self-perceived employability and actual employment among senior year students. Journal of Education and Work 30(8), 881–892. 10.1080/13639080.2017.1352083.

[r29] Keralis JM, Riggin-Pathak BL, Majeski T, Pathak BA, Foggia J, Cullinen KM, Rajagopal A and West HS (2018) Mapping the global health employment market: An analysis of global health jobs. BMC Public Health 18, 1–9. 10.1186/s12889-018-5195-1. PMC5830322 29486801

[r30] Khoury B and De Castro Pecanha V (2023) Transforming psychology education to include global mental health. Cambridge Prisms: Global Mental Health 10, E17. 10.1017/gmh.2023.11. 37854425 PMC10579692

[r31] Kidd SA, Kerman N, Cole D, Madan A, Muskat E, Raja S, Rallabandi S and McKenzie K (2015) Social entrepreneurship and mental health intervention: A literature review and scan of expert perspectives. International Journal of Mental Health and Addictions 13, 776–787. 10.1007/s11469-015-9575-9.

[r32] Kohrt BA, Marienfeld CB, Panter-Brick C, Tsai AC and Wainberg ML (2016) Global mental health: Five areas for value-driven training innovation. Academic Psychiatry 40, 650–658. 10.1007/s40596-016-0504-4. 26983416 PMC4938758

[r33] Lewis ME, Hartwell EE and Myhra LL (2018) Decolonizing mental health services for indigenous clients: A training program for mental health professionals. American Journal of Community Psychology 62(3–4), 330–339. 10.1002/ajcp.12288. 30561801

[r34] Marienfeld C, Hu X, Yang Y, Liu Z, Lasswell E and Rohrbaugh RM (2024) Educational partnerships: Addressing challenges in meeting trainee goals with established or new global mental health educational programs. In Acharya B and Becker AE (eds.), Global Mental Health Training and Practice. New York: Routledge, pp. 135–144. 10.4324/9781315160597-10.

[r35] Masten AS and Motti-Stefanidi F (2020) Multisystem resilience for children and youth in disaster: Reflections in the context of COVID-19. Adversity and Resilience Science 1(2), 95–106. 10.1007/s42844-020-00010-w. 32838305 PMC7314620

[r36] Matheson C, Robertson HD, Elliott AM, Iversen L and Murchie P (2016) Resilience of primary healthcare professionals working in challenging environments: A focus group study. British Journal of General Practice 66(648), e507–e515. 10.3399/bjgp16X685285. PMC4917054 27162205

[r37] Merritt C, Jack H, Mangezi W, Chibanda D and Abas M (2019) Positioning for success: Building capacity in academic competencies for early-career researchers in sub-Saharan Africa. Global Mental Health 6, E16. 10.1017/gmh.2019.14. 31391948 PMC6669964

[r38] Mishra A and Pandey N (2023) Global entrepreneurship in healthcare: A systematic literature review and bibliometric analysis. Global Business and Organizational Excellence 42, 9–21. 10.1002/joe.22193.

[r39] Morse JM (2015) Data were saturated … Qualitative Health Research 25(5), 587–588. 10.1177/1049732315576699. 25829508

[r40] Ng LC, Magidson JF, Hock RS, Joska JA, Fekadu A, Hanlon C, Galler JR, Safren SA, Borba CPC, Fricchione GL and Henderson DC (2016) Proposed training areas for global mental health researchers. Academic Psychiatry 40(4), 679–685. 10.1007/s40596-016-0518-y. 26976395 PMC4938780

[r41] Okewole H, Merritt C, Mangezi W, Mutiso V, Jack HE, Eley TC and Abas M (2020) Building career development skills for researchers: A qualitative study across four African countries. Annals of Global Health 86(1), 40. 32322538 10.5334/aogh.2759 PMC7164382

[r42] Perkins S, Nishimura H, Olatunde PF and Kalbarczyk A (2023) Educational approaches to teach students to address colonialism in global health: A scoping review. BMJ Global Health 8(4), e011610. 10.1136/bmjgh-2022-011610. PMC10106004 37055173

[r43] Pritchard J, Alavian S, Soogoor A, Bartels SA and Hall AK (2023) Global health competencies in postgraduate medical education: A scoping review and mapping to the CanMEDS physician competency framework. Canadian Medical Education Journal 14(1), 70–79. 10.36834/cmej.75275. PMC10042784 36998501

[r44] Rajabzadeh V, Burn E, Sajun SZ, Suzuki M, Bird VJ and Priebe S (2021) Understanding global mental health: A conceptual review. BMJ Global Health 6(3), e004631. 10.1136/bmjgh-2020-004631. PMC7993328 33758013

[r45] Rieckmann M (2012) Future-oriented higher education: Which key competencies should be fostered through university teaching and learning? Futures 44(2), 127–135. 10.1016/j.futures.2011.09.005.

[r46] Robson S and Wihlborg M (2019) Internationalisation of higher education: Impacts, challenges and future possibilities. European Educational Research Journal 18(2), 127–134. 10.1177/1474904119834779.

[r47] Rowthorn V and Olsen J (2014) All together now: Developing a team skills competency domain for global health education. Journal of Law, Medicine & Ethics 42(4), 550–563. Available at https://link.gale.com/apps/doc/A401904458/AONE?u=ustrath&sid=bookmark-AONE&xid=0be735cf (accessed 30 November 2023). 10.1111/jlme.12175 25565620

[r48] Schleiff M, Hansoti B, Akridge A, Dolive C, Hausner D, Kalbarczyk A, Pariyo G, Quinn TC, Rudy S and Bennett S (2020) Implementation of global health competencies: A scoping review on target audiences, levels, and pedagogy and assessment strategies. PLoS One 15(10), e0239917. 10.1371/journal.pone.0239917. 33002086 PMC7529249

[r49] Schleiff MJ, Mburugu PM, Cape J, Mwenesi R, Sirili N, Tackett S, Urassa DP, Hansoti B and Mashalla Y (2021) Training curriculum, skills, and competencies for global health leaders: Good practices and lessons learned. Annals of Global Health 87(1), 64. 10.5334/aogh.3212. 34307067 PMC8284497

[r50] Sridhar S, Alizadeh F, Ratner L, Russ CM, Sun SW, Sundberg MA and Rosman SL (2023) Learning to walk the walk: Incorporating praxis for decolonization in global health education. Global Public Health 18(1), 2193834. 10.1080/17441692.2023.2193834. 36989128

[r51] Suri H (2011) Purposeful sampling in qualitative research synthesis. Qualitative Research Journal 11(2), 63–75. 10.3316/QRJ1102063.

[r52] Tang C, Byrge C and Zhou J (2018) Creativity perspective on entrepreneurship. In Turcan R and Fraser N (eds.), The Palgrave Handbook of Multidisciplinary Perspectives on Entrepreneurship. Cham: Palgrave Macmillan. 10.1007/978-3-319-91611-8_5.

[r53] Thornicroft G, Cooper S, Bortel TV, Kakuma R and Lund C (2012) Capacity building in global mental health research. Harvard Review of Psychiatry 20(1), 13–24. 10.3109/10673229.2012.649117. 22335179 PMC3335140

[r54] Tran LT, Jung J, Unangst L and Marshall S (2023) New developments in internationalisation of higher education. Higher Education Research & Development 42(5), 1033–1041. 10.1080/07294360.2023.2216062.

[r55] Trowbridge J, Tan JY, Hussain S, Osman AEB and Di Ruggiero E (2022) Examining intersectoral action as an approach to implementing multistakeholder collaborations to achieve the sustainable development goals. International Journal of Public Health 67, 1604351. 10.3389/ijph.2022.1604351. 35652124 PMC9149775

[r56] Ventres WB (2019) Facilitating critical self-exploration by global health students. AMA Journal of Ethics 21(9), 749–758. 10.1001/amajethics.2019. 31550222

[r57] von Treuer KM and Reynolds N (2017) A competency model of psychology practice: Articulating complex skills and practices. Frontiers in Education 2, 54. 10.3389/feduc.2017.00054.

[r58] Wainberg ML, Scorza P, Shultz JM, Helpman L, Mootz JJ, Johnson KA, Neria Y, Bradford JME, Oquendo MA and Arbuckle MR (2017) Challenges and opportunities in global mental health: A research-to-practice perspective. Current Psychiatry Reports 19, 1–10. 10.1007/s11920-017-0780-z. 28425023 PMC5553319

[r59] White RG, Imperiale MG and Perera E (2016) The capabilities approach: Fostering contexts for enhancing mental health and wellbeing across the globe. Globalization and Health 12(1), 1–10. 10.1186/s12992-016-0150-3. 27150600 PMC4858826

[r60] White RG, Orr DM, Read UM and Jain S (2017) Situating global mental health: Sociocultural perspectives. In White RG, Jain S, Orr DMR and Read UM (eds.), The Palgrave Handbook of Sociocultural Perspectives on Global Mental Health. London: Springer Nature, pp. 1–27.

[r61] World Health Organization (2013) Building back better: Sustainable mental health care after emergencies. Available at https://www.who.int/publications/i/item/9789241564571 (accessed 31 August 2023).

[r62] World Health Organization (2017) mhGAP training manuals for the mhGAP intervention guide for mental, neurological and substance use disorders in non-specialized health settings, version 2.0 (for field testing). World Health Organization. Available at https://apps.who.int/iris/handle/10665/259161 (accessed 31 August 2023).

[r63] World Health Organization (2021) Bridging the divide: A guide to implementing the Humanitarian-Development-Peace Nexus for health. Available at https://iris.who.int/handle/10665/351260 (accessed 31 August 2023).

[r64] World Health Organization and UNICEF (2022) EQUIP - Ensuring quality in psychological support. Available at https://www.who.int/teams/mental-health-and-substance-use/treatment-care/equip-ensuring-quality-in-psychological-support (accessed 22 October 2023).

[r65] Wroe EB, McBain RK, Michaelis A, Dunbar EL, Hirschhorn LR and Cancedda C (2017) A novel scenario-based interview tool to evaluate nontechnical skills and competencies in global health delivery. Journal of Graduate Medical Education 9(4), 467–472. 10.4300/JGME-D-16-00848.1. 28824760 PMC5559242

[r66] Young T, Pakenham KI, Chapman CM and Edwards MR (2022) Predictors of mental health in aid workers: Meaning, resilience, and psychological flexibility as personal resources for increased well‐being and reduced distress. Disasters 46(4), 974–1006. 10.1111/disa.1251. 34617612

[r67] Zwanikken PA, Alexander L, Huong NT, Qian X, Valladares LM, Mohamed NA, Ying XH, Gonzalez-Robledo MC, Linh le C, Wadidi MS, Tahir H, Neupane S and Scherpbier A (2014) Validation of public health competencies and impact variables for low- and middle-income countries. BMC Public Health 14, 55. 10.1186/1471-2458-14-55. 24438672 PMC3899921

