# Supplementary material for: Identifying core global mental health professional competencies: A multi-sectoral perspective
Source: Glob Ment Health (Camb). 2024 Feb 22;11:e24. doi: 10.1017/gmh.2024.26 (PMC10988164; doi:10.1017/gmh.2024.26)
Supplement: Karadzhov et al. supplementary material [file S2054425124000268sup001.docx]

Supplementary Online Files

## Complete List of Competencies Identified in the Job Advertisement Analysis

*Note: Descriptions have only been included for the higher-level categories and sub-categories, which were developed by the researchers. Virtually all lower-level codes were directly taken from the job advertisements.*

| Name | Description |
| --- | --- |
| SECTOR | Skills, competencies and experience required in specific roles and sectors |
| ADVOCACY | A range of activities related to promoting the rights of, and empowering, service-users and other individuals |
| Engage in client advocacy and facilitate self-advocacy |  |
| Ensure service accessibility |  |
| Explain advocacy processes and outcomes |  |
| Raise awareness and challenge stigma |  |
| Represent clients who are unable to represent themselves |  |
| Support service-user involvement and empowerment |  |
| Use appropriate communication methods |  |
| Work with various stakeholders to promote fairness and equality |  |
| Deliver campaigns |  |
| Knowledge of relevant legislation |  |
| Service user involvement |  |
| POLICY DESIGN AND INFLUENCE | A range of tasks related to policy development such as policy analysis, stakeholder consultations and briefing papers preparation, |
| Contribution to policy development |  |
| Policy analysis |  |
| PROGRAMME DEVELOPMENT, IMPLEMENTATION AND EVALUATION | Designing, implementing and evaluating interventions and programmes across a range of settings |
| Programme evaluation and improvement |  |
| Collect data on programme outcomes |  |
| Explore ways to introduce new service improvements |  |
| Prepare progress reports |  |
| Understanding and experience of monitoring and evaluation practices and tools |  |
| Programme implementation and scaling-up |  |
| Contextualise and adapt global and country-specific materials |  |
| Document and disseminate good practice in programme design and delivery |  |
| Review and share programme-specific tools and resources |  |
| Support project implementation in line with local and global standards and guidelines |  |
| Support the scaling-up of EBIs and capacity-building in LMICs |  |
| Engage with local stakeholders and national governments to ensure adoption and sustainability of interventions |  |
| Training stakeholders |  |
| Programme planning and development |  |
| Adapting programmes and approaches to new contexts |  |
| Capacity and needs assessment |  |
| Experience adapting interventions |  |
| Integrate mental health services into primary care |  |
| Involve users in service design |  |
| SECTOR- AND COUNTRY-SPECIFIC KNOWLEDGE AND EXPERIENCE | Good familiarity and experience with relevant sectors, stakeholders, policies, legislation and low- and middle-income settings |
| Country- and region-specific experience |  |
| Experience of working in countries affected by conflict |  |
| Work experience in LMICs |  |
| Work experience outside one's own country |  |
| Policy and political knowledge |  |
| Knowledge of global frameworks and policies |  |
| Familiarity and experience with UN frameworks and systems |  |
| Understanding of country context |  |
| Understanding of government structures |  |
| Specialist sector knowledge |  |
| Knowledge of mental health policies and systems in LMICs |  |
| Knowledge of mental health services and legislation |  |
| Knowledge of pharmacy and other medical practices |  |
| Knowledge of social work systems, including child protection |  |
| Working with a range of stakeholders | Competencies and relevant experience in working with a wide range of stakeholders across sectors such as governmental officials, NGOs, external partners, local communities and user groups |
| Ability to engage, involve, persuade and motivate diverse stakeholders |  |
| Carry out stakeholder consultations |  |
| Coordinate and plan activities |  |
| Support stakeholder capacity-building |  |
| Demonstrate customer service excellence |  |
| Donor relations experience |  |
| Experience of working with governments, international agencies and NGOs |  |
| Knowledge and understanding of key stakeholders on global and regional levels |  |
| Partnership-building, incl. across sectors |  |
| SERVICE DELIVERY (CLINICAL, PSYCHOLOGICAL AND PSYCHOSOCIAL) | Direct, one-to-one delivery of clinical, psychological and psychosocial services |
| Build constructive therapeutic relationships |  |
| Carrying out psychological assessments |  |
| Case management services |  |
| Assist clients with developing life skills |  |
| Maintain high-quality case records |  |
| Manage caseloads effectively |  |
| Referral and signposting |  |
| Communicate in a skilled and sensitive manner |  |
| Dealing calmly, empathetically and effectively with people experiencing distress or people who are hostile |  |
| Develop and review support and safety plans |  |
| Develop treatment plans |  |
| Experience of working with vulnerable individuals |  |
| Facilitate client independence and self-management |  |
| Knowledge and experience of a range of interventions and care models |  |
| CAMHS |  |
| Counselling skills |  |
| Delivery services in community settings |  |
| Experience of psychosocial approaches |  |
| Knowledge and experience of MHPSS |  |
| Peer support approaches |  |
| Provide recovery-oriented care |  |
| Person-centred care |  |
| Client empowerment |  |
| Strengths-based care |  |
| Trauma-informed care |  |
| Work directly in a person-centred way |  |
| Knowledge of safeguarding practices |  |
| Liaison with health and non-health services |  |
| Maintain client confidentiality |  |
| Risk assessment |  |
| Use and promote evidence-based practices |  |
| Stay up-to-date with evidence-based practices |  |
| TEACHING SKILLS | Delivery of teaching, research supervision and pastoral support in higher education settings |
| SELF | Enduring personal characteristics, abilities and aptitudes |
| INTELLECTUAL ABILITIES | Cognitive abilities pertaining to analysing, synthesising and interpreting information and generating ideas and solutions |
| Analytical skills |  |
| Creativity and idea generation |  |
| Critical thinking |  |
| Problem-solving |  |
| Strategic thinker |  |
| PERSONAL RESOURCES | Traits and competencies that promote coping, resilience and thriving in the workplace |
| Accountability |  |
| Ambition, drive and motivation |  |
| Commitment to personal and professional development |  |
| Composure | The ability to work well under press while remaining composed and positive |
| Courage |  |
| Curiosity |  |
| Entrepreneurial mindset |  |
| Solution-oriented |  |
| Flexibility and adaptability | incl. being open-minded, embracing change and being open to new experiences and learning new skills |
| Passionate about global mental health |  |
| Positivity |  |
| Resilience and grit |  |
| Self-discipline |  |
| VALUES | A set of moral principles guiding one's career path and professional conduct |
| Care |  |
| Empathy and compassion |  |
| Fairness and inclusion |  |
| Awareness of racism and its impact |  |
| Humility |  |
| Integrity and honesty |  |
| Professionalism |  |
| Respect |  |
| Respect for diversity |  |
| Social justice commitment | A commitment to promoting the rights and inclusion of marginalised individuals and challenging injustice and unfairness |
| SKILLS - TRANSFERABLE | Transferable skills required across a wide range of professional settings. These include technical and soft skills. |
| INTERPERSONAL SKILLS | Abilities to communicate effectively in a range of formats and with a range of individuals to achieve a range of outcomes such as knowledge exchange, one-to-one support, relationship-building, influencing and people management |
| Communication and Interpersonal Skills |  |
| Conflict resolution |  |
| Influencing skills |  |
| Intercultural communication and collaboration |  |
| Leadership skills |  |
| Listening skills |  |
| Management and supervision of others |  |
| Non- judgemental and objective |  |
| Sensitivity and compassion |  |
| Teamwork and collaboration |  |
| Networking and relationship-building |  |
| Relationship-building skills |  |
| Training and mentorship |  |
| KNOWLEDGE DISSEMINATION AND EXCHANGE | The ability to communicate information, including research findings and policy analyses, to a variety of specialist and non-specialist audiences using various mediums |
| Adapt material for different audiences |  |
| Produce Lay Summaries |  |
| Contextualise and adapt global and country-specific materials |  |
| Create training materials |  |
| Data Visualisation |  |
| Design and facilitate workshops and knowledge exchange events |  |
| Policy briefs |  |
| Presentations |  |
| ORGANISATIONAL SKILLS | A set of skills essential to organising and prioritising workload effectively |
| Independence |  |
| Initiative |  |
| Priority-setting |  |
| Project management skills | Managing and supporting people; overseeing budgets and timelines; supporting project delivery; working with stakeholders to implement technical projects; knowledge of different project management techniques; and others. |
| Time management |  |
| RESEARCH SKILLS | Delivery and design of research, including knowledge of research methods and governance |
| Background of conducting research in culturally diverse contexts, particularly LMICs |  |
| Data collection, management and analysis | Competencies in specific research methods and techniques |
| Data analysis and management |  |
| Data analysis software skills |  |
| Statistical analysis |  |
| Statistical and computational programming |  |
| Data management and cleaning |  |
| Data collection methods and techniques | Methods and techniques for collecting quantitative and qualitative data |
| Experience of participant interviewing |  |
| Experience of participant recruitment |  |
| Participatory research methodologies |  |
| Peer research methods |  |
| Knowledge of research design and theory |  |
| Ethics knowledge and ethical practice |  |
| Ethical awareness |  |
| Cultural sensitivity |  |
| Political sensitivity |  |
| Self-awareness |  |
| Knowledge of GDPR |  |
| Obtain ethics approval |  |
| Literature reviews |  |
| Bibliographic software use |  |
| Literature review protocols |  |
| Research governance |  |
| Technical writing |  |
| Contribute to funding proposals |  |
| Publications in peer-reviewed scientific journals |  |
| Scientific writing |  |
| Use research to inform practice |  |
| SUBJECT | Working knowledge of theories, concepts, frameworks and principles relevant to global mental health research and practice. |
| Core GMH knowledge |  |
| Capacity-building approaches |  |
| Cultural adaptation of interventions |  |
| Knowledge of adapting interventions |  |
| Cultural context and mental health |  |
| Familiarity with GMH tools and guidelines |  |
| Humanitarian support principles |  |
| Implementation science |  |
| Social determinants of health |  |
| Expertise in related disciplines |  |
| Anthropology |  |
| Disability studies |  |
| Epidemiology |  |
| Human rights |  |
| International development |  |
| Public health |  |
| Social work |  |
| Up-to-date knowledge of psychology |  |
| Mental health and psychosocial interventions |  |
| CAMHS |  |
| Community-based interventions |  |
| Evidence-based interventions |  |
| MHPSS |  |
| Personal recovery principles |  |
| Person-centred care |  |
| Suicide prevention |  |
| Trauma-informed care |  |
