# Supplementary material for: Identifying core global mental health professional competencies: A multi-sectoral perspective
Source: Glob Ment Health (Camb). 2024 Feb 22;11:e24. doi: 10.1017/gmh.2024.26 (PMC10988164; doi:10.1017/gmh.2024.26)
Supplement: Peer review history

# Editor report: Recommendation: Identifying core global mental health professional competencies: A multi-sectoral perspective — R0/PR2

**DOI:** 10.1017/gmh.2024.26.pr2

**Affiliations:** Faculty of Business, Technology, and Education , Ilia State University, Georgia

No accompanying comment.

# Editor report: Decision: Identifying core global mental health professional competencies: A multi-sectoral perspective — R0/PR3

**DOI:** 10.1017/gmh.2024.26.pr3

**Affiliations:** Johns Hopkins University Bloomberg School of Public Health, United States

No accompanying comment.

# Editor report: Recommendation: Identifying core global mental health professional competencies: A multi-sectoral perspective — R1/PR5

**DOI:** 10.1017/gmh.2024.26.pr5

**Affiliations:** Faculty of Business, Technology, and Education , Ilia State University, Georgia

No accompanying comment.

# Editor report: Decision: Identifying core global mental health professional competencies: A multi-sectoral perspective — R1/PR6

**DOI:** 10.1017/gmh.2024.26.pr6

**Affiliations:** Johns Hopkins University Bloomberg School of Public Health, United States

No accompanying comment.
